# A Novel Alginate-Based Delivery System for the Prevention and Treatment of Pressure-Overload Induced Heart Failure

**DOI:** 10.3389/fphar.2020.602952

**Published:** 2021-02-02

**Authors:** Ambrish Kumar, Marwa Belhaj, Donald J. DiPette, Jay D. Potts

**Affiliations:** ^1^ Department of Cell Biology and Anatomy, School of Medicine, University of South Carolina, Columbia, SC, United States; ^2^ Department of Internal Medicine, School of Medicine, University of South Carolina, Columbia, SC, United States

**Keywords:** alginate microcapsules, calcitonin gene-related peptide, cardiovascular diseases, congestive heart failure, drug delivery system, neuropeptide, pressure-overload heart failure, transverse aortic constriction

## Abstract

**Background:** α-CGRP (alpha-calcitonin gene related peptide) is a cardioprotective neuropeptide. Our recent study demonstrated that the administration of native α-CGRP, using osmotic mini-pumps, protected against transverse aortic constriction (TAC) pressure-induced heart failure in mice. However, the short half-life of peptides and the non-applicability of osmotic pumps in humans limits the use of α-CGRP as a therapeutic agent for heart failure (HF). Here, we sought to comprehensively study a novel α-CGRP delivery system using alginate microcapsules to determine its bioavailability *in vivo* and to test for cardioprotective effects in HF mice.

**Methods:** Native α-CGRP filled alginate microcapsules (200 µm diameter) were prepared using an electrospray method. The prepared alginate-α-CGRP microcapsules were incubated with rat cardiac H9c2 cells, mouse cardiac HL-1 cells, and human umbilical vein endothelial cells (HUVECs), and the cytotoxicity of the alginate-α-CGRP microcapsules was measured by a trypan-blue cell viability assay and a calcium dye fluorescent based assay. The efficacy of the alginate-α-CGRP microcapsules was tested in a TAC-pressure overload mouse model of heart failure. Male C57BL6 mice were divided into four groups: sham, sham-alginate-α-CGRP, TAC-only, and TAC-alginate-α-CGRP, and the TAC procedure was performed in the TAC-only and TAC-alginate-α-CGRP groups of mice to induce pressure-overload heart failure. After 2 or 15 days post-TAC, alginate-α-CGRP microcapsules (containing an α-CGRP dose of 6 mg/kg/mouse) were administered subcutaneously on alternate days, for 28 days, and echocardiography was performed weekly. After 28 days of peptide delivery, the mice were sacrificed and their hearts were collected for histological and biochemical analyses.

**Results:** Our *in vitro* cell culture assays showed that alginate-α-CGRP microcapsules did not affect the viability of the cell lines tested. The alginate-α-CGRP microcapsules released their peptides for an extended period of time. Our echocardiography, biochemical, and histology data from HF mice demonstrated that the administration of alginate-α-CGRP microcapsules significantly improved all cardiac parameters examined in TAC-mice. When compared to sham mice, TAC significantly decreased cardiac functions (as determined by fraction shortening and ejection fraction) and markedly increased heart and lung weight, left ventricle (LV) cardiac cell size, cardiac apoptosis, and oxidative stress. In contrast, the administration of alginate-α-CGRP microcapsules significantly attenuated the increased heart and lung weight, LV cardiac cell size, apoptosis, and oxidative stress in TAC mice.

**Conclusion:** Our results demonstrate that the encapsulation of α-CGRP in an alginate polymer is an effective strategy to improve peptide bioavailability in plasma and increase the duration of the therapeutic effect of the peptide throughout the treatment period. Furthermore, alginate mediates α-CGRP delivery, either prior to the onset or after the initiation of the symptom progression of pressure-overload, improves cardiac function, and protects hearts against pressure-induced HF.

## Introduction

Alpha-calcitonin gene related peptide (α-CGRP), a 37 amino acid neuropeptide, is considered the most potent vasodilator discovered to date, and possesses positive chronotropic and inotropic effects (Brain et al., 1985; Supowit et al., 1995; Al-Rubaiee et al., 2013). Extensive studies from our laboratory and others have established a protective function for α-CGRP in a variety of cardiovascular diseases, including heart failure, myocardial infarction, and experimental hypertension (Gangula et al., 1997; Katki et al., 2001; Supowit et al., 2005; Chai et al., 2006; Huang et al., 2008; Li et al., 2013; Russell et al., 2014; Kumar et al., 2019a). In addition, α-CGRP delivery lowers blood pressure (BP) in normal as well as hypertensive animals and humans (DiPette et al., 1987; DiPette et al., 1989; Dubois-Rande et al., 1992; Supowit et al., 1993). Using α-CGRP knock-out (KO) mice, our laboratory showed that, in comparison with wild-type mice, KO mice exhibited greater cardiac hypertrophy, cardiac dilation and dysfunction, cardiac fibrosis, and mortality when subjected to transverse aortic constriction (TAC) pressure-overload induced heart failure (Li et al., 2013). Our recent study demonstrated that long-term exogenous delivery of native α-CGRP using osmotic mini-pumps attenuated the adverse effects of TAC pressure-overload induced heart failure in wild-type mice (Kumar et al., 2019b). the application of native α-CGRP for 28 days preserved cardiac function, and reduced apoptotic cell death, fibrosis, and oxidative stress in TAC left ventricles (LVs), thus confirming the cardioprotective function of α-CGRP in congestive heart failure. Similarly, two other studies confirmed that an infusion of either native α-CGRP (Skaria et al., 2019) or an α-CGRP-agonist analog (an acylated form of α-CGRP with a half-life of t_1/2_ = ∼7 h) (Aubdool et al., 2017) significantly improved cardiac function in rodent models of hypertension and heart failure. These lines of evidence further confirm that α-CGRP, either native or its derivative, is a promising drug candidate to treat hypertension, heart failure, or other cardiovascular diseases. However, the short half-life of α-CGRP (t_1/2_ = ∼5.5 min in human plasma) (Maassen Van Den Brink et al., 2016) and the non-applicability of implanted osmotic pumps in humans limits the use of α-CGRP as a therapeutic agent for long-term treatment. Therefore, novel delivery systems that could increase the bioavailability of the peptide in the serum are needed.

Alginate polymers have garnered favor recently as an FDA approved novel drug carrier. There are ongoing clinical trials on alginate-based drug delivery formulations (Lee and Mooney, 2012). Alginate is a water-soluble linear polysaccharide isolated from brown algae. Structurally, it is an unbranched polyanionic polysaccharides of 1–4 linked α-L-guluronic acid and β-D-mannuronic acid. As the alginate polymer is stable at a wide range of temperatures (0–100°C), non-toxic, and biocompatible, a variety of substrates including peptides, DNA, antibodies, proteins, and cells have been used for encapsulation (Gu et al., 2004; Zhang et al., 2011; Moore et al., 2013a). Our laboratory routinely uses alginate-based drug delivery technology to encapsulate various proteins, inhibitors, and cells (Moore et al., 2013a; Moore et al., 2013b; Annamalai et al., 2018) to treat both corneal wounds in diabetic rats and macular degeneration in mouse models (Moore et al., 2014; Belhaj et al., 2020).

The aim of the present study was to develop a novel alginate-based drug delivery system applicable for the long-term sustained release of α-CGRP in humans. We used an electrospray method to encapsulate α-CGRP in alginate microcapsules and tested the efficacy of the microcapsules in the prevention and treatment of TAC pressure-overload induced heart failure. Our results show that subcutaneous administration of alginate-α-CGRP microcapsules immediately after TAC surgery and prior to the onset of symptoms significantly protects hearts at the physiological and cellular level. Thus, our state-of-the-art technology to encapsulate α-CGRP and its delivery through alginate microcapsules offers new options to benefit people suffering from cardiovascular diseases.

## Materials and Methods

### Preparation of Alginate-α-CGRP Microcapsules

An electrospray method was used to prepare α-CGRP encapsulated alginate microcapsules of 200 µm diameter (Moore et al., 2013a). Briefly, a 2% alginic acid solution (high mannuronic acid content and low viscosity; Millipore Sigma, St. Louis, MO) was prepared in sterile triple-distilled water and filtered through a 0.2 μm syringe filter. A stock solution of 2 mg/ml of mouse native α-CGRP (GenScript United States Inc, Piscataway, NJ) was prepared in a sterile 0.9% NaCl saline solution and further sterilized through a 0.2 µm syringe filter. Five hundred micrograms of prepared α-CGRP was mixed with 1 ml of 2% alginic acid and passed through a positively charged syringe at a constant rate under a high voltage current into the 150 mM CaCl_2_ gelling solution to make calcium-coated alginate-α-CGRP microcapsules. Alginate-only microcapsules were prepared under similar conditions. The prepared microcapsules were washed 4–5 times with sterile triple-distilled water for 5 min each to remove excess CaCl_2_, then the α-CGRP filled microcapsules were finally suspended in 500 µl of sterile triple-distilled water. The release of the peptide from the alginate-α-CGRP microcapsules was confirmed by an *in vitro* α-CGRP release assay. Briefly, 250 μl of the supernatant from the prepared microcapsules was collected at various time points and stored at 4 °C, and the volume was made up each time with sterile water. Peptide concentration in the collected supernatant was quantitated with a MicroBCA protein assay kit (Pierce/ThermoScientific, Waltham, MA) using mouse α-CGRP as the standard. The supernatant collected from alginate-only microcapsules was used as the control. The final absorbance was measured at 450 nm using a Spectramax Plus-384 microplate reader (Molecular Devices, Sunnyvale, CA) and plotted.

### Pressure-Overload Heart Failure Mouse Model

Eight-week-old male C57/BL6 mice (Charles River Laboratories, Wilmington, MA) were maintained on a 12 h light/12 h dark cycle with free access to standard food and water. Mice were allowed to acclimate for one week after shipment. Animal protocols were approved by the University of South Carolina-Institutional Animal Care and Use Committee following the National Institutes of Health, United States guidelines.

Pressure-overload heart failure in mice was induced by a transverse aortic constriction (TAC) procedure (Li et al., 2013; Kumar et al., 2019b). Briefly, the chests of anesthetized mice (under 1–1.5% isoflurane) were opened through the suprasternal notch, and a 7-0 suture (Ethicon prolene polypropylene blue) was passed under the aortic arch between the left common carotid and innominate arteries. The suture was tied around both the aorta and a 27-gauge needle. After placing a knot, the needle was removed. This procedure yielded 70–80% aortic constriction. The chest was closed using a 6-0 silk suture and mice were allowed to recover. Sham-operated mice underwent an identical procedure except for the aortic constriction. Two days post-surgery, the mice were divided into four groups: sham (*n* = 8), sham-alginate-CGRP (*n* = 7), TAC-only (*n* = 7), and TAC-alginate-CGRP (*n* = 8). In the sham-alginate-CGRP and TAC-alginate-CGRP groups of mice, α-CGRP-encapsulated alginate microcapsules (containing an α-CGRP dose of 6 mg/kg/mouse) were injected subcutaneously into the flank region of mice on alternate days, for 28 days. At the end of the experiment (day 28 of α-CGRP delivery), mice from all the groups were weighed and euthanized. The wet weight of hearts and lungs were measured and photographed. The basal portion of the hearts was fixed in 4% paraformaldehyde/PBS (pH 7.4) for histochemistry, while an apical portion was snap frozen in liquid N_2_ and stored at −80°C for biochemical analyses.

Another set of animal studies (CGRP-treatment study) were conducted in which alginate-α-CGRP microcapsule delivery was started 15 days post-TAC. The mice were divided into four groups: sham (*n* = 5), sham-alginate-CGRP (*n* = 4), TAC-only (*n* = 4), and TAC-alginate-CGRP (*n* = 4), and the TAC procedure was performed in the TAC-only and TAC-alginate-CGRP groups of mice. Fifteen days post-TAC, alginate-α-CGRP microcapsules (containing an α-CGRP dose of 6 mg/kg/mouse) were injected subcutaneously into the flank region of mice on alternate days, for 28 days. The treatment regime for both studies is found in the supplementary material (Supplementary Figure S2; Scheme 1 for 2-day post-TAC alginate-α-CGRP microcapsules delivery, and Scheme 2 for 15 days post-TAC alginate-α-CGRP microcapsules delivery). At the conclusion of the study (day 28), the mice were euthanized, and tissues were collected as discussed before. The clinical criteria needed to remove an animal from the study were a >15% weight loss, reduced physical activity, guarding, ruffled fur, and abnormal sensitivity. Mice who died during the TAC procedure and within 24 h after TAC (due to surgery complications) were not counted in the study. Two days after the TAC procedure, mice who underwent aortic constriction were randomized into the TAC-only and TAC-alginate-αCGRP groups, while sham-operated mice were randomized into the sham-only and sham-alginate-αCGRP groups. After starting alginate-α-CGRP microcapsule administration, mice from all the groups survived until the end of the study, and we did not observe any adverse effects in the mice.

### Transthoracic Echocardiography

A Vevo 3100 High-Resolution Imaging System (VisualSonics Inc, Toronto, Canada) was used to perform the echocardiography in the mice (Kumar et al., 2019b). Mice were sedated under 2% isoflurane and their heart rate was maintained at 450 ± 20 beats per minute. Short axis B- and M-mode 2D echocardiograms were recorded through the anterior and posterior LV walls at the level of the papillary muscle. Fractional shortening (FS) and ejection fraction (EF) were calculated by the VisualSonics Measurement Software.

### Blood Pressure Measurement

The blood pressure (BP) of sham and treatment mice was recorded by a non-invasive tail-cuff method using the MC4000 BP Analysis System (Hatteras Instruments, Cary, NC). To reduce stress-induced changes, mice were trained at least three consecutive days prior to the baseline BP recording. On the day of the BP measurement, mice were normalized in the recording room for at least 1 h and kept on the instrument platform for 5 min to bring animal body temperature to the instrument temperature. After measuring the baseline BP (designated as 0 h), alginate microcapsules (with or without α-CGRP) were administered subcutaneously into the flank region of the mice and BP was again recorded.

### Western Blotting

Total protein from the LVs was extracted using a RIPA cell lysis buffer (Cell Signaling Technology, Danvers, MA), and protein concentration was measured by a BCA protein assay kit (Pierce) (Kumar et al., 2011). An equal amount of the protein samples (40 μg) were mixed with 5× Laemmli sample buffer, heated at 95°C for 10 min, and separated on SDS-polyacrylamide gel followed by the transfer to the PVDF membrane at 100 V for 3 h in a cold room. The membrane was blocked with 10% non-fat dry milk prepared in TBST (20 mM Tris-Cl, pH 7.4; 150 mM NaCl with 0.1% Tween-20) for 4 h at room temperature and further incubated in primary antibodies overnight at 4°C. Protein signals were detected by adding HRP-conjugated secondary antibodies (Bio-Rad Laboratories, Hercules, CA) for 2 h at room temperature and using a Clarity Western Detection Kit (Bio-Rad). The primary antibodies used were cleaved caspase-3 and β-actin (Cell Signaling Technology).

### Immunohistochemistry

Paraformaldehyde-fixed paraffin-embedded LV sections (5 μm) were deparaffinized and rehydrated with xylene and graded ethanol (100, 95, and 70%), respectively, and boiled in 10 mM sodium citrate buffer (pH 6.0) for 30 min for antigen retrieval (Kumar et al., 2014). After permeabilization with 0.2% Triton X-100/PBS for 10 min, the LV sections were blocked with 10% IgG-free-BSA/PBS (Jackson ImmunoResearch Laboratories, West Grove, PA) and incubated with primary antibodies overnight at 4°C. Alexafluor-488 or Alexafluor-546 conjugated secondary antibodies (Invitrogen, Carlsbad, CA) were added to detect protein signals. After mounting with antifade-mounting media (Vector Laboratories, Burlingame, CA), the tissue sections were examined under a Nikon-E600 fluorescence microscope (Nikon, Japan). The primary antibodies used were: cleaved caspase-3 (Cell Signaling) and anti-4-hydroxy-2-nonenal (4-HNE; Abcam Inc, Cambridge, MA). DAPI (4′, 6-diamidino-2-phenylindole; Sigma) was used to stain the nuclei.

Hematoxylin and eosin (H&E) staining, Texas Red-X conjugated wheat germ agglutinin staining (WGA staining; Invitrogen), and Masson’s trichrome-collagen staining (PolyScientific, Bay Shore, NY) were performed using the vendors’ protocols to measure LV cardiac cell size, cardiomyocyte cross-sectional area, and fibrosis, respectively, and quantitated using the NIH-ImageJ software (NIH, United States).

### Cell Lines and *In Vitro* Toxicity Assays

#### Trypan-Blue Cell Viability Assay

The rat cardiac H9C2 cells were grown at 37°C in a humidified incubator with 5% CO_2_ in a complete culture medium (containing DMEM supplemented with 10% fetal bovine serum, FBS, 4.5 gm/liter D-glucose, and 1x penicillin/streptomycin). Human umbilical vein endothelial cells (HUVECs) were maintained in an F-12K medium containing 10% FBS, 0.1 mg/ml heparin, 30 mg/l endothelial cell growth supplement (Sigma), and 1x penicillin/streptomycin at 37°C in a humidified incubator with 5% CO_2_. The viability of the H9C2 cells and HUVECs in the presence of alginate-α-CGRP microcapsules was determined by a trypan-blue assay (Sigma). Briefly, stock solution of rat/mouse α-CGRP (1 mg/ml) was prepared in sterile 0.9% NaCl solution and filter sterilized through a 0.2 µm syringe filter. H9C2 cells and HUVECs, grown in complete culture medium, were treated with alginate-only, α-CGRP, or alginate-α-CGRP microcapsules. Following treatments, the cells were photographed under a phase-contrast microscope to examine the cell morphology. After seven days of treatment, the cells were trypsinized and counted by a hemocytometer using the trypan-blue exclusion method.

#### Calcium Dye Fluorescent Based Assay

The mouse cardiac muscle cell line, HL-1 cells, were grown on gelatin and fibronectin-coated cell culture flasks in Claycomb Basal Medium (Sigma) supplemented with 10% FBS, 0.1 mM norepinephrine in ascorbic acid, 2 mMl-glutamine, and 1× penicillin/streptomycin soln. HL-1 cells were maintained at 37°C in a humidified incubator with 5% CO_2_, and the cell culture media was exchanged every day.

A cell permeant calcium dye fluorescent based assay was performed in gelatin and a fibronectin-coated 24-well culture plate to observe the viability (beating phenotype) of the HL-1 cells. Briefly, at 100% cell confluency, 500 μl of 5 μM cell permeable calcium indicator dye Fluo-4AM (Invitrogen) in HEPES-buffered Hanks’ solution was added in each well followed by incubation at 37°C for 1 h in a humidified incubator. After incubation, the cells were washed in Hanks’ solution and 500 μl Hanks’ solution was added. Cells were immediately viewed using the EVOS FL auto2 microscope (Invitrogen). Using the 10× objective setting, spontaneous contraction of the HL-1 cells was video recorded (considered as 0 h). A volume of 500 μl Hanks’ solution containing 10 μM alginate-α-CGRP microcapsules was added and further video recorded.

### Enzymatic Activity Assay

A GSH-Glo Glutathione assay kit (Promega) was used to measure the total glutathione (GSH) content in the LVs following the vendor’s instructions. Briefly, 10 mg of LV heart tissue was homogenized in 1× PBS containing 2 mM EDTA, centrifuged at 12,000 rpm for 15 min at 4°C, and the supernatant was collected. A total of 50 μl of GSH-Glo Reagent was mixed with 50 μl of tissue extract (10 μg) and incubated for 30 min at RT. Next, 100 μl of luciferin detection reagent was added and incubated for an additional 15 min at RT. The signal was measured using a Turner 20/20 luminometer (Promega).

### Statistical Analysis

Comparisons were made among the groups using a Student t-test and one-way ANOVA followed by a Tukey-Kramer ad hoc test (GraphPad software, La Jolla, CA). A *p* value <0.05 was considered significant.

## Results

### Encapsulation of α-CGRP and Release From Alginate Microcapsules

**FIGURE 1:**
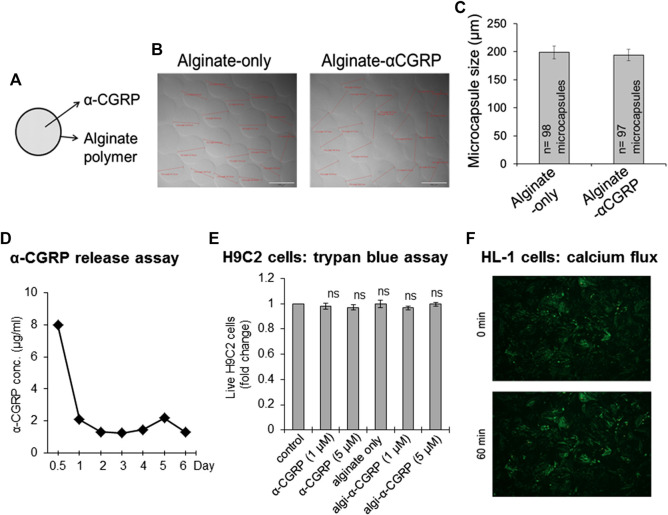
Preparation of the alginate-α-CGRP microcapsules. **(A–C)** An electrospray method was used to encapsulate α-CGRP in an alginate polymer. The prepared alginate-only and alginate-α-CGRP microcapsules were photographed **(B)** and their size was measured and plotted **(C)**. **(D)** An *in vitro* α-CGRP release assay showing the amount of α-CGRP released in the supernatant from alginate-α-CGRP microcapsules. **(E)** Bar diagram showing the number of live H9C2 cells, as measured by trypan-blue cell viability assay, after 7 days incubation with different concentrations of α-CGRP-alone, empty-alginate microcapsules, and alginate-α-CGRP microcapsules. ns, not significant compared to control. **(F)** The viability of mouse HL-1 cardiac cells in the presence of alginate-α-CGRP microcapsules (10 µM) was determined by an *in vitro* calcium flux fluorescence assay as discussed in the methods section. HL-1 cells stained with Fluo-4AM dye were imaged using EVOS auto-F2 microscope at 0 and 60 min after the addition of alginate-α-CGRP microcapsules (10 µM).

α-CGRP was encapsulated using an electrospray method with the following experimental conditions to prepare alginate-α-CGRP microcapsules of 200 μm diameter size. α-CGRP (500 μg from a stock 2 mg/ml soln) was mixed with 1 ml of 2% alginic acid solution and loaded to a 3 ml syringe attached to a high-voltage generator. A beaker filled with 30 ml of ionic gelling bath solution containing 150 mM CaCl_2_ was placed below the syringe pump and the distance between the syringe needle to the CaCl_2_ gelling bath solution was kept at 7 mm. The alginate-α-CGRP mixture was passed through the positively charged syringe needle at a constant rate (flow rate: 60 mm/h) under a high voltage current (6 KV) into the negatively charged CaCl_2_ gelling bath, creating spherical Ca^+2^-coated alginate-α-CGRP microcapsules of 200 µm diameter. We also prepared alginate-only microcapsules of similar size. The prepared microcapsules were photographed and the size of the microcapsules was measured. The calculated average size of the alginate-only and alginate-α-CGRP microcapsules was 198.84 ± 11.34 and 194.23 ± 10.08 μm, respectively (Figures 1A–C). The release of α-CGRP from the prepared alginate-α-CGRP microcapsules was determined by an *in vitro* α-CGRP release assay. Figure 1D shows that presence of α-CGRP was detected in the supernatant for up to 6 days indicating that alginate-α-CGRP microcapsules released peptides over an extended period of time.

### Alginate-α-CGRP Microcapsules Exhibit No Cytotoxicity

It is crucial in determining the effect of the release of α-CGRP on the heart to show that cardiac muscle cells are not altered by the addition of the capsules. To that end we used two different cardiac cell lines: rat H9C2 cells and mouse HL-1 cells, and two different cell viability assays: a trypan-blue exclusion assay and a calcium dye fluorescent based assay, to determine the cytotoxicity of the prepared alginate-α-CGRP microcapsules. H9C2 cells were grown in complete culture medium in the presence of alginate-α-CGRP microcapsules (1 or 5 μM). After seven days of incubation with the capsules, a trypan-blue exclusion assay was carried out. Results from the assay demonstrated that the viability of the H9C2 cells was similar among the treatment groups when compared to control-untreated cells (ns = non-significant compared to control, Figure 1E).

The viability of the mouse HL-1 cardiac cells in the presence of the alginate-α-CGRP microcapsules was determined using an *in vitro* calcium flux fluorescence assay. HL-1 cells stained with Fluo-4AM dye were video recorded to monitor both the beating phenotype and calcium fluxes inside the cell and imaged using an EVOS auto-F2 microscope. After taking images at the basal time point (0 min), the alginate-α-CGRP microcapsules (containing 10 µM α-CGRP) were added and were further video recorded. Images (Figure 1F) and videos (Supplementary Figure S1) taken at time points 0 and 60 min after the addition of alginate-α-CGRP microcapsules demonstrated that the alginate-α-CGRP microcapsules (10 µM) did not affect the myocyte contraction of the HL-1 cells. These data support our statement that alginate-α-CGRP microcapsules do not exhibit cytotoxicity against the cardiac cell lines tested. In addition, the incubation of alginate-α-CGRP microcapsules for 7 days did not affect the growth of HUVE cells and the number of live cells among the treated and untreated control remained same (ns = non-significant compared to control, Supplementary Figure S3).

### Alginate-α-CGRP Microcapsules Reduce Blood Pressure in Mice

**FIGURE 2:**
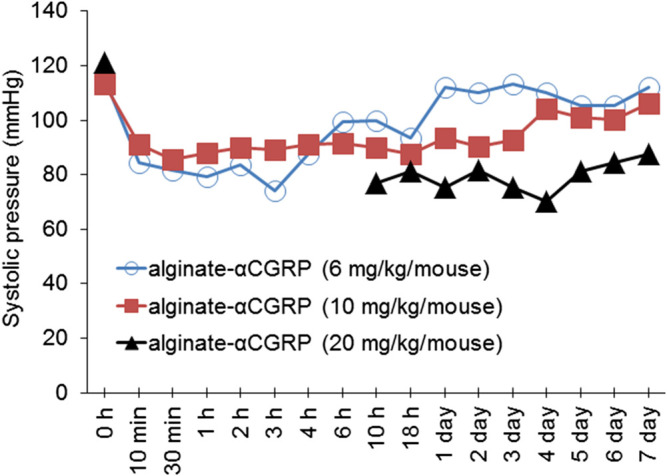
Effect of alginate-α-CGRP microcapsules on blood pressure in wild-type mice. Graph showing the systolic pressure, as measured by tail-cuff blood pressure method, after subcutaneous injection of various concentrations of alginate-α-CGRP microcapsules in mice.

α-CGRP is well-known to reduce BP (Kee et al., 2018), thus we set out to confirm the biological activity of released α-CGRP from alginate-α-CGRP microcapsules by measuring changes in BP. Three different doses of alginate microcapsules were injected subcutaneously in mice (2 mice/dose) and systolic pressure was monitored at various time points. Data shown in Figure 2 demonstrate that alginate-α-CGRP microcapsules containing α-CGRP doses (mg/kg/mouse) of 6 mg/kg and 10 mg/kg lowered the systolic pressure for up to 18 h and 3 days, respectively, after which time the BP returned to basal level. Subcutaneous administration of alginate-α-CGRP microcapsules containing α-CGRP doses of 20 mg/kg/mouse drastically reduced the BP for the first 6 h so much so that it could not be recognized by the instrument but by 10 h it registered as low and remained below basal levels over the next 7 days. However, subcutaneous administration of an equal amount of alginate-only microcapsules had no effect on the BP in mice (data not shown). These data confirm that α-CGRP is being released from the alginate microcapsules under *in vivo* conditions and that the released α-CGRP remains biologically active for an extended period of time.

### Alginate-α-CGRP Microcapsule Delivery Improves Cardiac Functions in TAC Mice

**FIGURE 3:**
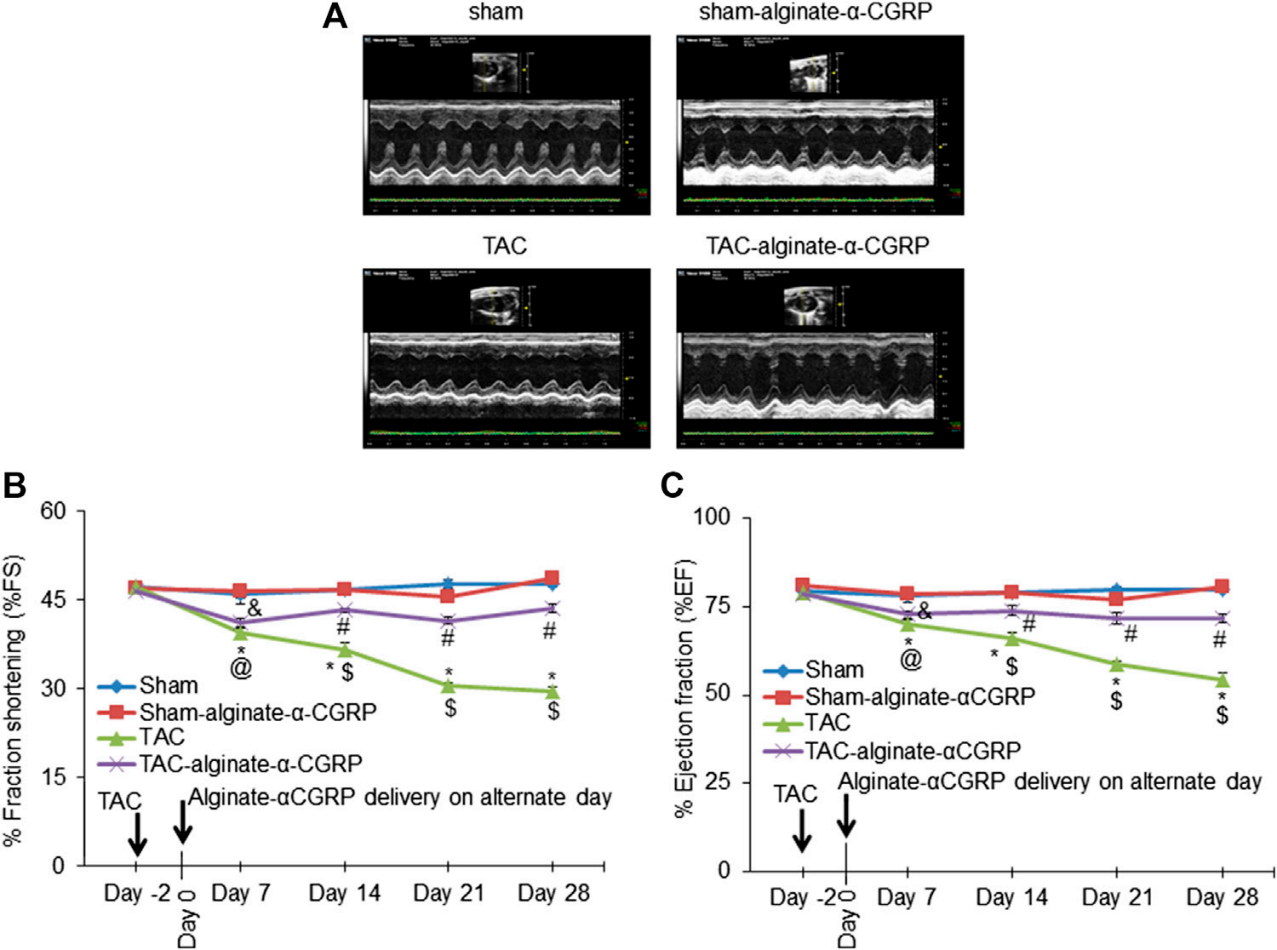
Alginate-α-CGRP microcapsules delivery in two-day post-TAC mice improves cardiac functions. **(A)** Representative echocardiograms showing short axis B- and M-mode 2D echocardiography performed after 28 days of delivery of alginate-α-CGRP microcapsules in sham and TAC mice. Percentage fractional shortening (FS) and ejection fraction (EF) was calculated at various time points and plotted **(B,C)**. *p < 0.05, TAC vs sham at the same time point; #*p* < 0.05, TAC-alginate-α-CGRP vs sham at the same time point; $*p* < 0.05, TAC vs TAC-alginate-α-CGRP at the same time point. @*p* < 0.05, TAC day 7 vs TAC day-2; and &*p* < 0.05, TAC-alginate-α-CGRP on day 7 vs TAC-alginate-α-CGRP on day-2.

Our previous studies demonstrated that continual α-CGRP administration following TAC surgery showed a cardioprotective capability (Kumar et al., 2019b). Therefore to determine if the alginate-α-CGRP microcapsules also had a cardioprotective effect, B- and M-mode 2D electrocardiography was performed on every 7^th^ day, up to day 28, following subcutaneous administration of alginate-α-CGRP microcapsules containing an α-CGRP dose of 6 mg/kg/mouse (Figures 3A–C). Over the course of the experiment, LV systolic function was assessed by measuring both the % fraction shortening (FS; Figure 3B) and ejection fraction (EF; Figure 3C). Both measures were significantly decreased as expected in the TAC mice when compared to the sham mice. However, repeated administration of the alginate-α-CGRP microcapsules starting 2 days after TAC surgery showed significant preservation of both cardiac parameters in treated TAC mice.

### α-CGRP Administration Attenuates Cardiac Hypertrophy and Fibrosis in TAC Mice

**FIGURE 4:**
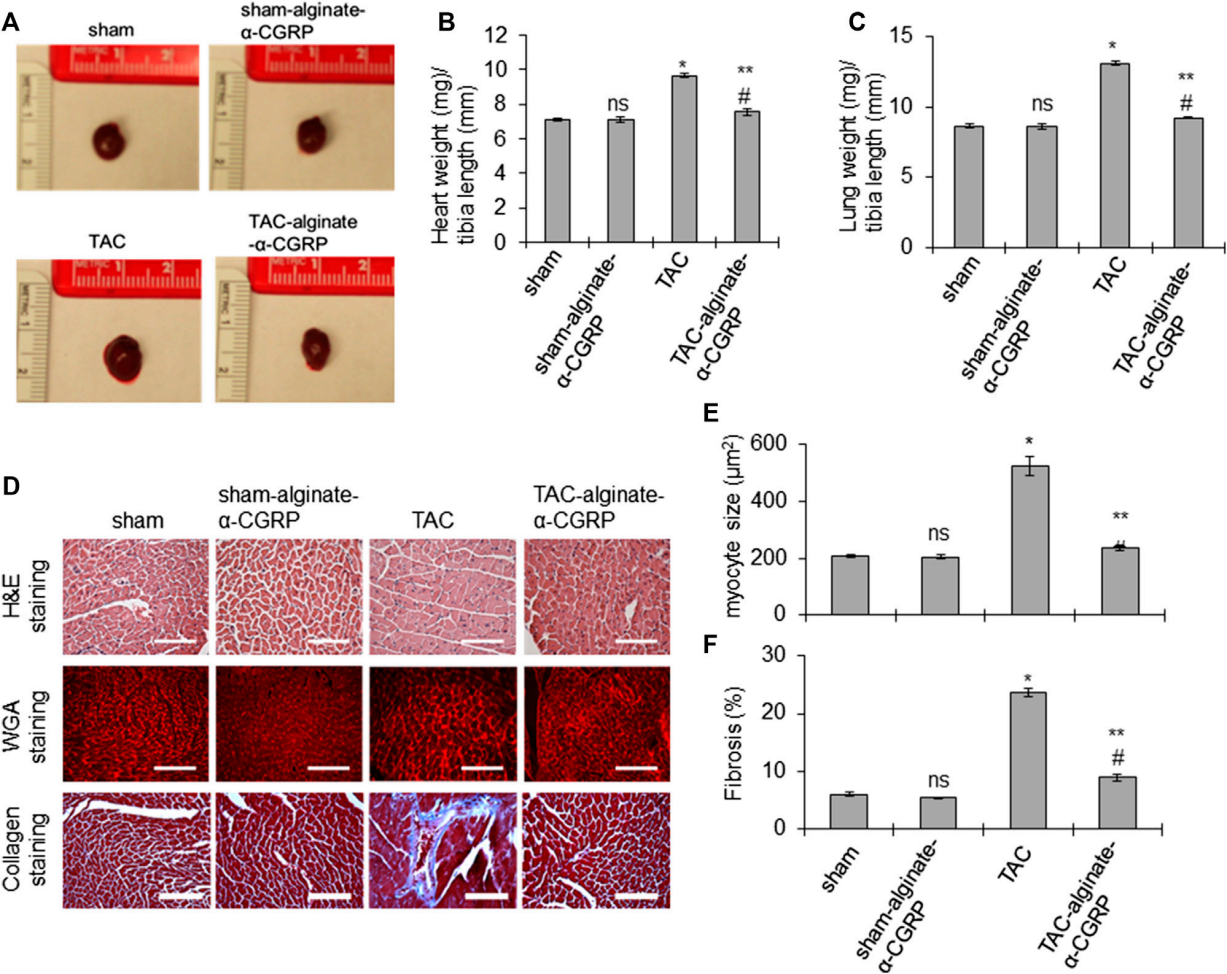
Alginate-α-CGRP microcapsules delivery in two-day post-TAC mice reduces TAC-induced cardiac hypertrophy and fibrosis. **(A)** Representative images showing the size of the hearts after 28 days of delivery of alginate-α-CGRP microcapsules. **(B**,**C)** Bar diagrams showing the ratio of wet heart weight/tibia length, and wet lung weight/tibia length. **(D)** The paraffin-embedded LV sections were stained with H&E, WGA stain, and trichrome-collagen stain. Scale bar, 100 μm. WGA stained sections were used to measure cardiomyocyte size in LVs by the NIH-ImageJ software and plotted **(E)**. LV collagen content, an indicator of fibrosis, was quantitated by the NIH-ImageJ software and plotted **(F)**. Values were expressed as the mean ± SEM. *p < 0.05, TAC vs sham; **p < 0.05, TAC-alginate-α-CGRP vs TAC; #*p* > 0.05, TAC-alginate-α-CGRP vs sham; ns, non-significant compared to sham.

In order to determine if the cardiac cellular damage was also attenuated by alginate-α-CGRP microcapsule treatment, gross and histological measurements were taken of hearts from all of the groups. At the conclusion of the experiment, all groups, treated and sham, were sacrificed. The hearts and lungs were isolated, photographed, and the ratio of wet heart weight to tibia length and wet lung weight to tibia length were measured as indices of LV hypertrophy and dilation and pulmonary congestion (Figures 4A–C). The representative photographs and bar diagrams in Figures 4A,B show that hearts from TAC mice were larger than hearts from the sham mice (*p < 0.05, TAC-only vs sham). Additionally, hearts from mice treated with alginate-α-CGRP microcapsules was significantly smaller than TAC (**p < 0.05, TAC-alginate-α-CGRP vs TAC) and comparable to sham hearts (#*p >* 0.05, TAC-alginate-α-CGRP vs sham-only; Figures 4A,B). Similarly, the calculated mean lung weight/tibia length was significantly greater in TAC mice compared to sham mice (*p < 0.05, TAC vs sham) while the increase in lung weight/tibia length after TAC was significantly reduced by α-CGRP administration (**p < 0.05, TAC-alginate-α-CGRP vs TAC-only; Figure 4C). The lung weight between the TAC-alginate-α-CGRP and sham groups of mice was not significantly different (#*p >* 0.05, TAC-alginate-α-CGRP vs sham). The heart size and the ratios of heart weight/tibia length and lung weight/tibia length among the sham-alginate-α-CGRP mice and sham-only mice appeared nearly identical (ns, sham-alginate-α-CGRP vs sham-only; Figures 4A–C).

To determine the effect of alginate-α-CGRP microcapsule treatment on cardiac myocyte size, H&E staining and wheat germ agglutinin (WGA) staining was performed (Figure 4D). As expected, the TAC procedure markedly increased myocyte size in the LVs (*p < 0.05, TAC vs sham, Figure 4E). However, LV myocyte size in the TAC-alginate-α-CGRP group was significantly decreased compared to TAC-only mice and was almost identical to sham-only mice (**p < 0.05, TAC-alginate-α-CGRP vs TAC-only; and #*p* > 0.05, TAC-alginate-α-CGRP vs sham). Treatment with alginate-α-CGRP microcapsules did not affect LV cardiomyocyte size in sham-alginate-α-CGRP mice when compared to sham LV (ns = nonsignificant vs sham). Likewise, when compared to sham, TAC surgery significantly increased LV fibrosis which was decreased with α-CGRP administration in TAC mice (*p < 0.05, TAC vs sham; **p < 0.05, TAC-alginate-α-CGRP vs TAC; #*p* < 0.05, TAC-alginate-α-CGRP vs sham; Figures 4D,F).

### α-CGRP Administration Reduces Apoptosis and Oxidative Stress in TAC LVs

**FIGURE 5:**
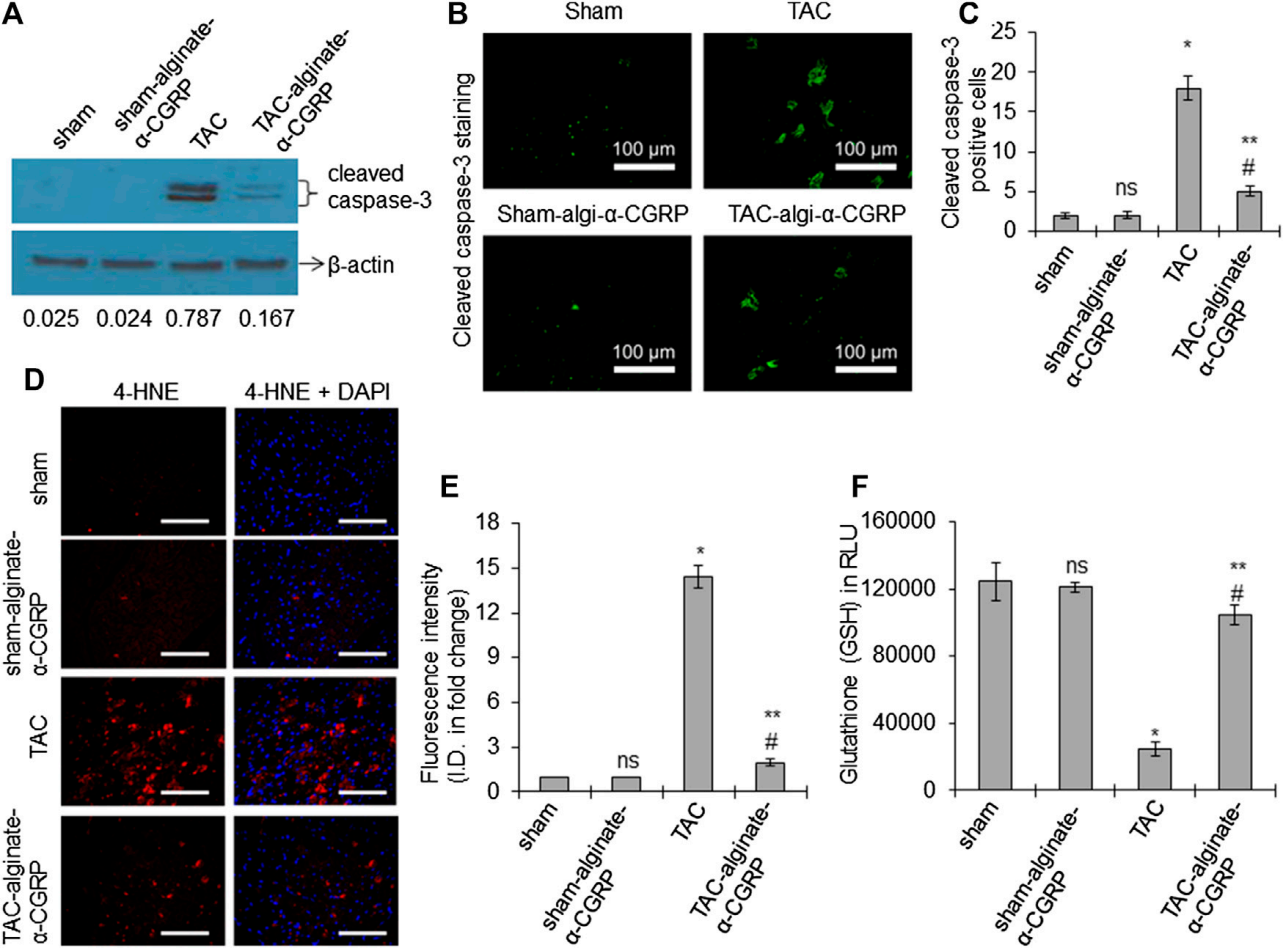
Alginate-α-CGRP microcapsules delivery in two-day post-TAC mice attenuates increased LV apoptosis and oxidative stress in TAC mice. **(A)** Western blot showing the level of cleaved caspase-3 protein in LVs from sham, sham-alginate-α-CGRP, TAC, and TAC-alginate-α-CGRP. β-actin was used as control. The density of the protein bands were quantitated by NIH-ImageJ and cleaved caspase-3/β-actin band density ratio was reported. **(B)** Representative fluorescence images showing cleaved caspase-3 staining (green) to detect apoptosis in the LV sections. Scale = 100 μm. Cleaved caspase-3 positive cells (green) were counted and plotted as the mean ± SEM **(C)**. **(D,E)** Fluorescence images showing 4-HNE staining (a marker of lipid peroxidation) in the paraffin-embedded LV sections. DAPI was used to stain nuclei. Scale = 100 μm. The fluorescence intensity of 4-HNE (red) was quantitated by the NIH-ImageJ software and plotted as the mean ± SEM. I.D. = integrated density. **(F)** Bar diagrams showing glutathione (GSH) level in the LVs. Values were expressed as the mean ± SEM and *p* < 0.05 was considered significant. *p < 0.05, TAC vs sham; **p < 0.05, TAC-alginate-α-CGRP vs TAC; #*p* > 0.05, TAC-alginate-α-CGRP vs sham; ns = not-significant compared to sham.

Our previous studies showed that following TAC, there is an increase in cell death and an elevation in oxidative stress markers (Li et al., 2013; Kumar et al., 2019b). We therefore set out to determine if α-CGRP administration could mitigate these responses. Western blot analysis for the presence of apoptosis markers demonstrated that cleaved caspsase-3 (a marker of apoptotic cell death) was significantly higher in TAC LVs compared to sham LVs, and alginate-α-CGRP microcapsules administration significantly reduced cleaved caspsase-3 levels to those observed in sham LVs (Figure 5A). Similarly, the number of cleaved caspase-3 positive cells (green) were higher in TAC LVs when compared to the sham LVs (*p < 0.05, TAC vs sham; Figures 5B,C). Similarly, when we analyzed the number of cleaved caspase-3 positive cells, we determined that it was significantly lower in the TAC-alginate-α-CGRP LVs to TAC LVs and comparable to that of sham LVs (**p < 0.05, TAC-alginate-α-CGRP vs TAC; #*p* < 0.05, TAC-alginate-α-CGRP vs sham; Figures 5B,C).

We also examined the hearts for 4-HNE, a marker of oxidative stress-induced lipid-peroxidation. Sections of LVs were imaged and its immunofluorescence quantitated. We observed that TAC induced pressure-overload markedly increased the formation of HNE-adduct in the TAC LV (*p < 0.05, TAC vs sham; Figures 5D,E), and α-CGRP administration significantly reduced the intensity of the signal of 4-HNE in the TAC LV and was comparable to their sham counterpart (**p < 0.05, TAC-alginate-α-CGRP vs TAC; #*p* < 0.05, TAC-alginate-α-CGRP vs sham). Figure 5F showed that the total glutathione level was significantly reduced in the TAC LVs (*p < 0.05, TAC vs sham) while significantly restored by treatment of alginate-α-CGRP microcapsules (**p < 0.05, TAC-alginate-α-CGRP vs TAC; #*p* < 0.05, TAC-alginate-α-CGRP vs sham). All of the oxidative stress parameters in the sham-alginate-α-CGRP LVs were comparable with sham LVs (ns = non-significant compared to sham; Figures 5D–F). These results suggest that α-CGRP delivery through alginate microcapsules protected cardiac cells from pressure-overload induced apoptosis and oxidative stress.

### Alginate-α-CGRP Microcapsule Administration Improves Cardiac Function in 15 days Post-TAC Mice

**FIGURE 6:**
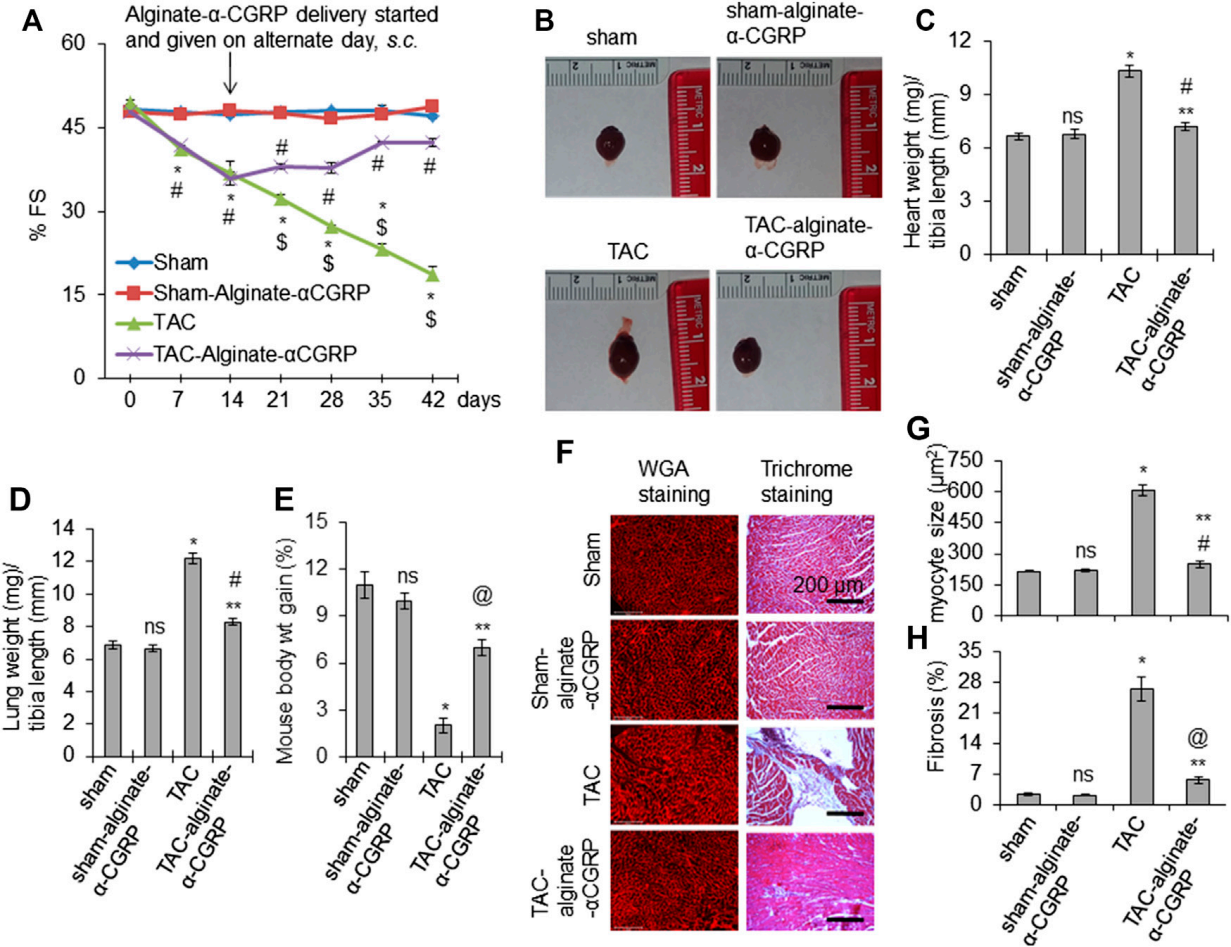
Alginate-α-CGRP microcapsule administration in 15-days post-TAC mice improves cardiac functions. **(A)** Graph showing % FS in sham, sham-alginate-α-CGRP, TAC-only, and TAC-alginate-α-CGRP groups of mice. After 15 days of TAC, alginate-α-CGRP microcapsules containing α-CGRP doses of 6 mg/kg/mouse were injected on alternate days, until day 28. Echocardiography was performed at different time points and % FS was plotted as mean ± SEM. *p < 0.05, TAC vs sham at the same time point; #*p* < 0.05, TAC-alginate-α-CGRP vs sham at the same time point; $*p* < 0.05, TAC vs TAC-alginate-α-CGRP at the same time point. **(B)** Representative images showing the size of hearts after 28 days of delivery of alginate-α-CGRP microcapsules. Ratio of wet heart weight/tibia length was plotted as mean ± SEM **(C)**. **(D)** Bar diagram showing ratio of wet lung weight/tibia length as mean ± SEM. **(E)** Bar diagram showing mice weight gain (in percentage) during the course of the experiment as mean ± SEM. *p* < 0.05 was considered significant. *p < 0.05, TAC vs sham; **p < 0.05, TAC-alginate-α-CGRP vs TAC; #*p* > 0.05, TAC-alginate-α-CGRP vs sham; ^@^ *p* < 0.05, TAC-alginate-α-CGRP vs sham; ns, not-significant compared to sham. **(F)** Representative histology images showing the size of cardiomyocytes (WGA staining) and level of fibrosis (trichrome-collagen staining) in the LVs from different groups of mice. Cardiomyocyte size **(G)** and % fibrosis **(H)** in LVs were quantitated using the NIH-ImageJ software and plotted as mean ± SEM. *p* value <0.05 was considered significant. *p < 0.05, TAC vs sham; **p < 0.05, TAC-alginate-α-CGRP vs TAC; #*p* > 0.05, TAC-alginate-α-CGRP vs sham; ^@^ *p* < 0.05, TAC-alginate-α-CGRP vs sham; ns = not-significant compared to sham.

Our results from these experiments demonstrated that α-CGRP microcapsule delivery, beginning 2 days post-TAC, protected mice against adverse pressure-induced cardiac effects. We next wanted to determine if our alginate-α-CGRP microcapsules could ameliorate these effects after the progression of heart failure had already begun. This would move our studies from a preventive approach to an actual treatment approach. To address this, we again performed TAC surgery in mice, and then 15 days after TAC, alginate-α-CGRP microcapsules (containing an α-CGRP dose of 6 mg/kg/mouse) were administered *s.c*. on alternate days for an additional 28 days. Day 15 was chosen as the time point when all deleterious measures of heart failure would be present in mice following TAC surgery. Echocardiogram data showed the usual result that TAC significantly reduced cardiac fraction shortening (FS) (*p < 0.05, TAC vs sham). What was exciting was that alginate-α-CGRP microcapsules administration attenuated the reduction in FS following 28 days of treatment. The FS in TAC-alginate-α-CGRP mice was significantly improved compared to TAC mice and was comparable with that of sham mice ($*p* < 0.05, TAC vs TAC-alginate-α-CGRP at the same time point) (Figure 6A). When compared to TAC mice, the wet heart wt and lung wt in TAC-alginate-α-CGRP mice was significantly lower indicating that α-CGRP delivery significantly inhibited cardiac hypertrophy and pulmonary edema in TAC mice (Figures 6B–D). During the length of the experiment, the TAC group of mice gained only 2% body wt. while sham, sham-alginate-α-CGRP, and TAC-alginate-α-CGRP group of mice gained (in %) 11, 10, and seven body wt, respectively, indicating that α-CGRP improved body gain in TAC mice (Figure 6E). Moreover, the administration of alginate-α-CGRP microcapsules starting at day 15, significantly attenuated the increased size of cardiomyocytes (Figures 6F,G) and fibrosis **(**as determined by collagen content after Masson’s trichrome collagen staining; Figures 6F,H) in TAC LVs after 28 days of treatment. Although α-CGRP concentration used in the present study significantly inhibited fibrosis in TAC LVs, it did not reduce the level to that observed in sham LVs (Figure 6H). Our CGRP-treatment study demonstrated, for the first time, that α-CGRP alginate microcapsule administration beginning 15 days post-TAC protected hearts both at the physiological and pathological levels and reversed the deleterious effects of pressure overload in the heart.

## Discussion

Studies from our laboratory and other research groups established that α-CGRP deletion makes the heart more vulnerable to heart failure, hypertension, myocardial infarction, and cardiac and cerebral ischemia (Bowers et al., 2005; Supowit et al., 2005; Li et al., 2013; Schebesch et al., 2013; Kumar et al., 2019a). Moreover, exogenous delivery of α-CGRP peptides show benefits against cardiac diseases. In patients with stable angina pectoris, intracoronary infusion of α-CGRP delayed the onset of myocardial ischemia (Mair et al., 1990). Also, in patients with congestive heart failure, an acute intravenous infusion of α-CGRP improved myocardial contractility and thus improved cardiac functions (Gennari et al., 1990). Similarly, an infusion of α-CGRP in patients with heart failure decreased systemic arterial pressure (Dubois-Rande et al., 1992). Our previous rodent study confirmed that long-term administration of α-CGRP, through osmotic mini-pumps, significantly preserved the heart at the functional and anatomical levels in TAC pressure-overload mice (Kumar et al., 2019b). A similar study using α-CGRP KO mice presented data that support our findings on the cardioprotective role of α-CGRP in cardiac diseases and showed that α-CGRP delivery through osmotic mini-pumps corrected the adverse effects of hypertension in these KO mice (Skaria et al., 2019). However, the low bioavailability of the native peptide in human plasma (t_1/2_ = ∼5.5 min) makes it difficult to use α-CGRP as a therapeutic agent in a long-term treatment regime. Moreover, the applicability of an osmotic mini-pump as a peptide delivery system is also not feasible in humans.

The present study demonstrated that using an alginate polymer as a drug carrier for α-CGRP was effective in ameliorating pressure-overload induced heart failure. Moreover, cell apoptosis and oxidative stress that accompany worsening heart failure was reduced by the treatment with alginate-α-CGRP microcapsules. Another important finding of the study is that alginate-α-CGRP microcapsules subcutaneously administered every other day in pressure-overload heart failure mice, improved myocardial function by restoring both FS and EF, hallmarks of increasing heart failure and moreover, attenuated increased apoptotic cell death and oxidative stress in TAC LVs.

Previously, it has been shown that intravenous injections of α-CGRP significantly decreases mean arterial pressure (MAP) in a dose-dependent fashion in both normal and spontaneously hypertensive rats, however, MAP returns to a normal baseline after 20 min of injection in both groups of rats (Ando et al., 1990). Our findings demonstrated that subcutaneous administration of alginate-α-CGRP microcapsules containing α-CGRP doses of 6 mg/kg/mouse and 10 mg/kg/mouse lowered the systolic pressure for 18 h and 3 days, respectively. Moreover, our results indicate that the addition of alginate-α-CGRP microcapsules extends the release of peptides, and the released α-CGRP remains biologically active for extended periods of time.

Another novel and exciting finding of the present study is that when alginate microcapsules were administered starting at 15-days post-TAC mice there was an immediate reversal of symptoms. This was similar to the ability of α-CGRP filled alginate microcapsules to significantly protect hearts when administered immediately after surgery. Also similar to early administration, treatment started at 15 days post-TAC was able to reverse all of the parameters of heart failure examined including, cardiac hypertrophy, apoptosis, cardiac function, and fibrosis. This is the first demonstration that the addition of α-CGRP just prior to the onset of symptoms could quickly reverse the damage that is observed with TAC-induced heart failure. The encapsulated peptides remained biologically active *in vivo* as released α-CGRP from subcutaneously administered alginate-α-CGRP microcapsules lowered the BP, an inherent property of α-CGRP, in mice (Figure 2). Moreover, alginate-α-CGRP microcapsule formulation is non-toxic to cardiac cells and HUVE cells (Figures 1E,F, and Supplementary Figure S3).

Furthermore, alginate microcapsules are very stable at room temperature and remain intact even after 15 months in media (data not shown). Our laboratory has also established that alginate microcapsules can undergo freeze-thaw cycles as well as can be lyophilized without compromising the integrity of the microcapsules (data not shown). The lyophilized form of alginate microcapsules immediately swell and regain their shape when rehydrated in distilled water. Consequently, alginate-α-CGRP microcapsules show promise as a flexible and diverse system to use to deliver α-CGRP in the future.

In summary, the present study demonstrated that an alginate microcapsule-based delivery system was an effective strategy to improve α-CGRP bioavailability in heart failure, and thus, increase the duration of the therapeutic effect of the peptide throughout the treatment period. In addition, the observed cardioprotective effects of alginate-α-CGRP microcapsules were present either administering prior to symptom onset (i.e., CGRP-prevention study) or at 15 days post-TAC when symptoms are just beginning (i.e., CGRP-treatment study). Thus, the developed alginate-α-CGRP microcapsule administration can be effective in the prevention and represents a new treatment option for pressure-induced heart failure. The success of this novel drug delivery technology will have the potential to dramatically change conventional drug therapies presently used to treat the failing heart.

## Data Availability Statement

The original contributions presented in the study are included in the article/Supplementary Material, further inquiries can be directed to the corresponding author.

## Ethics Statement

The animal study was reviewed and approved by USC IACUC.

## Author Contributions

AK, DD, and JP designed the study. MB prepared microcapsules. AK performed animal studies and conducted *in vitro*, histological, and biochemical assays, and analyzed data. AK, DD, and JP drafted and wrote the manuscript.

## Funding

This work is supported by the Distinguished Health Sciences Professorship of the University of South Carolina (DD.), the National Science Foundation EPSCoR Program (JP), and is partially supported by ASPIRE grants (ASPIRE-1 grant to JDP, and ASPIRE-1 Track-IIB grant to AK) from the Office of the Vice President for Research at the University of South Carolina.

## Conflict of Interest

The authors declare that the research was conducted in the absence of any commercial or financial relationships that could be construed as a potential conflict of interest.

## Supplementary Material

/SUPPLEMENTARY VIDEOS | Videos showing contraction of mouse HL-1 cardiac cells at time point 0 h (File name: HL1A2_0 hr) and 60 min (File name: HL1A2_60 min) after addition of alginate-α-CGRP microcapsules (10 μM) in in vitro calcium flux fluorescence assay.

## Abbreviations

α-CGRP, alpha-calcitonin gene-related peptide; BP, blood pressure, EF, ejection fraction; FS, fractional shortening; GSH, glutathione; 4-HNE, 4-hydroxynonenal; KO, knock-out; LV, left ventricle; s.c., subcutaneous; TAC, transverse aortic constriction; WGA, wheat germ agglutinin.
