# Supplementary material for: A Novel Alginate-Based Delivery System for the Prevention and Treatment of Pressure-Overload Induced Heart Failure
Source: Front Pharmacol. 2021 Feb 2;11:602952. doi: 10.3389/fphar.2020.602952 (PMC7884831; doi:10.3389/fphar.2020.602952)
Supplement: Supplementary file 1 [file presentation1.pptx]

## Slide 1
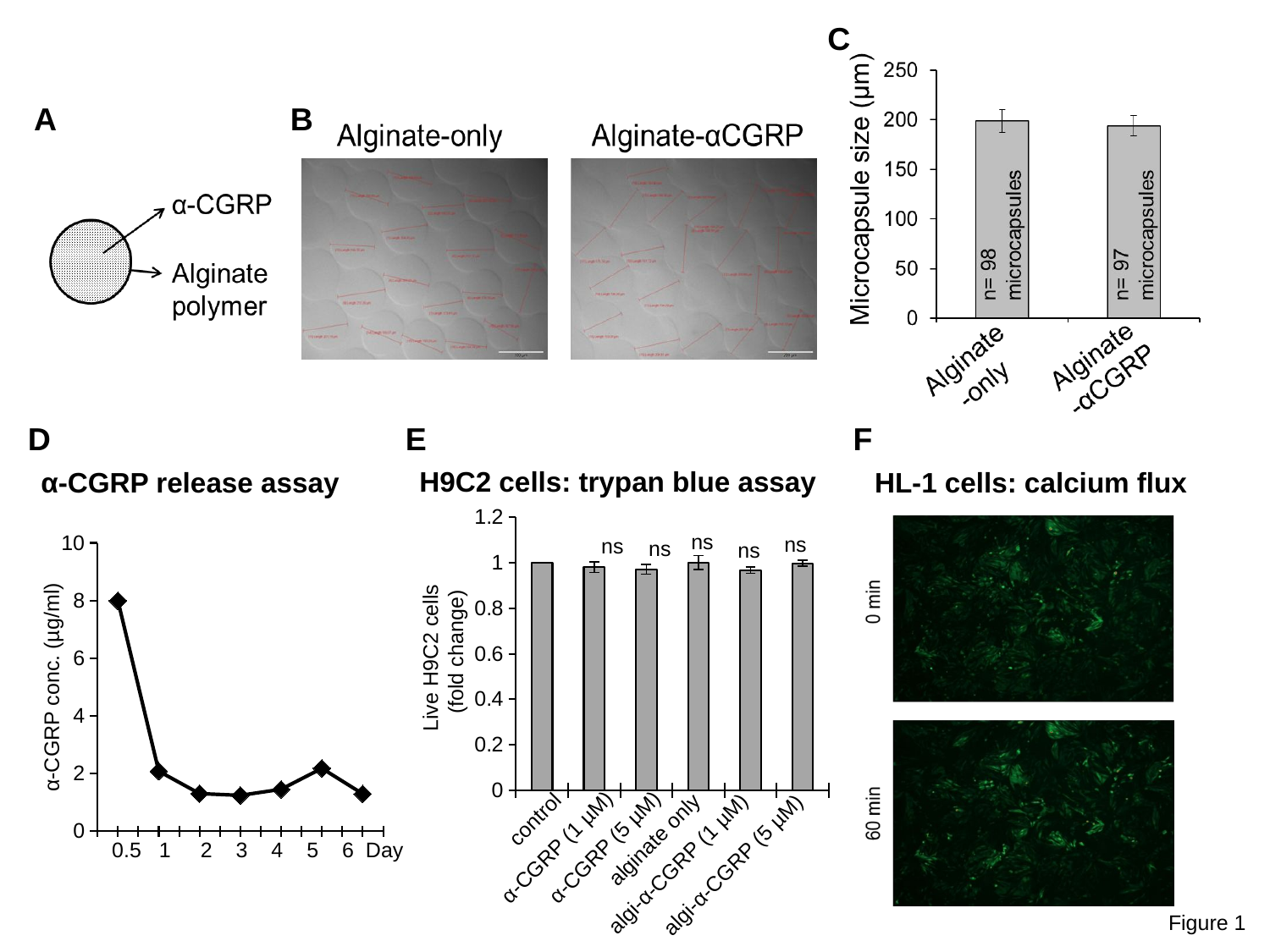

C
A
B
D
α-CGRP release assay
### Chart
| Category | |
|---|---|α-CGRP conc. (µg/ml)
0.5 1 2 3 4 5 6 Day
E
H9C2 cells: trypan blue assay
### Chart
| Category | |
|---|---|ns
ns
ns
ns
ns
Live H9C2 cells
 (fold change)
control
alginate only
α-CGRP (1 µM)
α-CGRP (5 µM)
algi-α-CGRP (1 µM)
algi-α-CGRP (5 µM)
F
HL-1 cells: calcium flux
Figure 1

## Slide 2
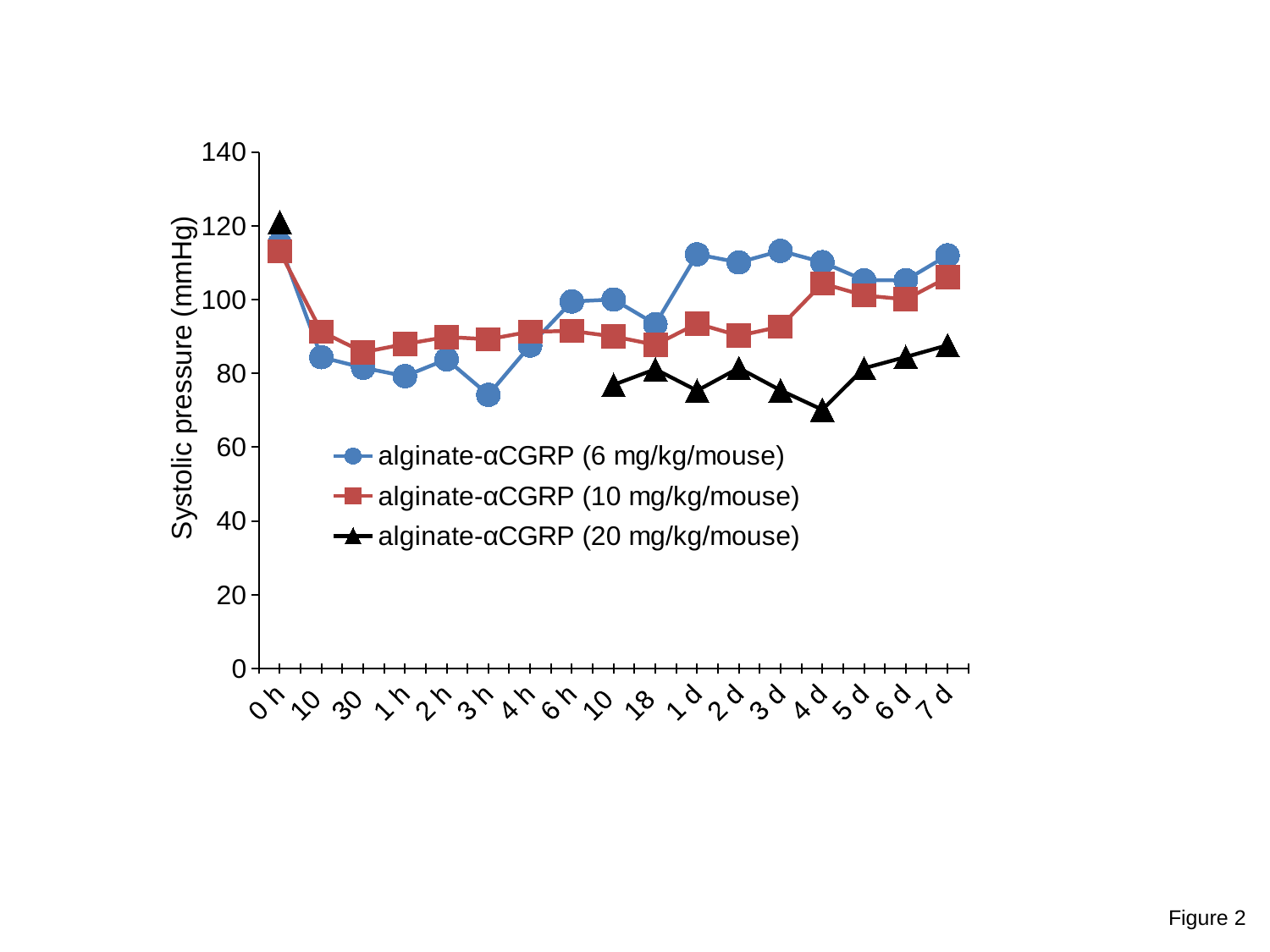

### Chart
| Category | alginate-αCGRP (6 mg/kg/mouse) | alginate-αCGRP (10 mg/kg/mouse) | alginate-αCGRP (20 mg/kg/mouse) |
|---|---|---|---|
| 0 h | 115.16666666666666 | 113.1 | 121.0 |
| 10 min | 84.38888888888889 | 91.33333333333333 | None |
| 30 min | 81.54166666666666 | 85.75 | None |
| 1 h | 79.25 | 87.94444444444444 | None |
| 2 h | 83.8 | 89.87 | None |
| 3 h | 74.2 | 89.25 | None |
| 4 h | 87.54700854700855 | 91.3 | None |
| 6 h | 99.48181818181817 | 91.49 | None |
| 10 h | 100.03333333333333 | 90.0 | 76.91666666666667 |
| 18 h | 93.35227272727272 | 87.75 | 81.14285714285714 |
| 1 day | 112.26648351648352 | 93.5 | 75.33333333333333 |
| 2 day | 110.06666666666666 | 90.205 | 81.46666666666667 |
| 3 day | 113.2076923076923 | 92.69 | 75.41666666666667 |
| 4 day | 110.13986013986013 | 104.36500000000001 | 70.16666666666667 |
| 5 day | 105.26785714285714 | 101.13 | 81.4 |
| 6 day | 105.26785714285714 | 100.07 | 84.46 |
| 7 day | 112.0 | 106.0 | 87.64285714285714 |Systolic pressure (mmHg)
Figure 2

## Slide 3
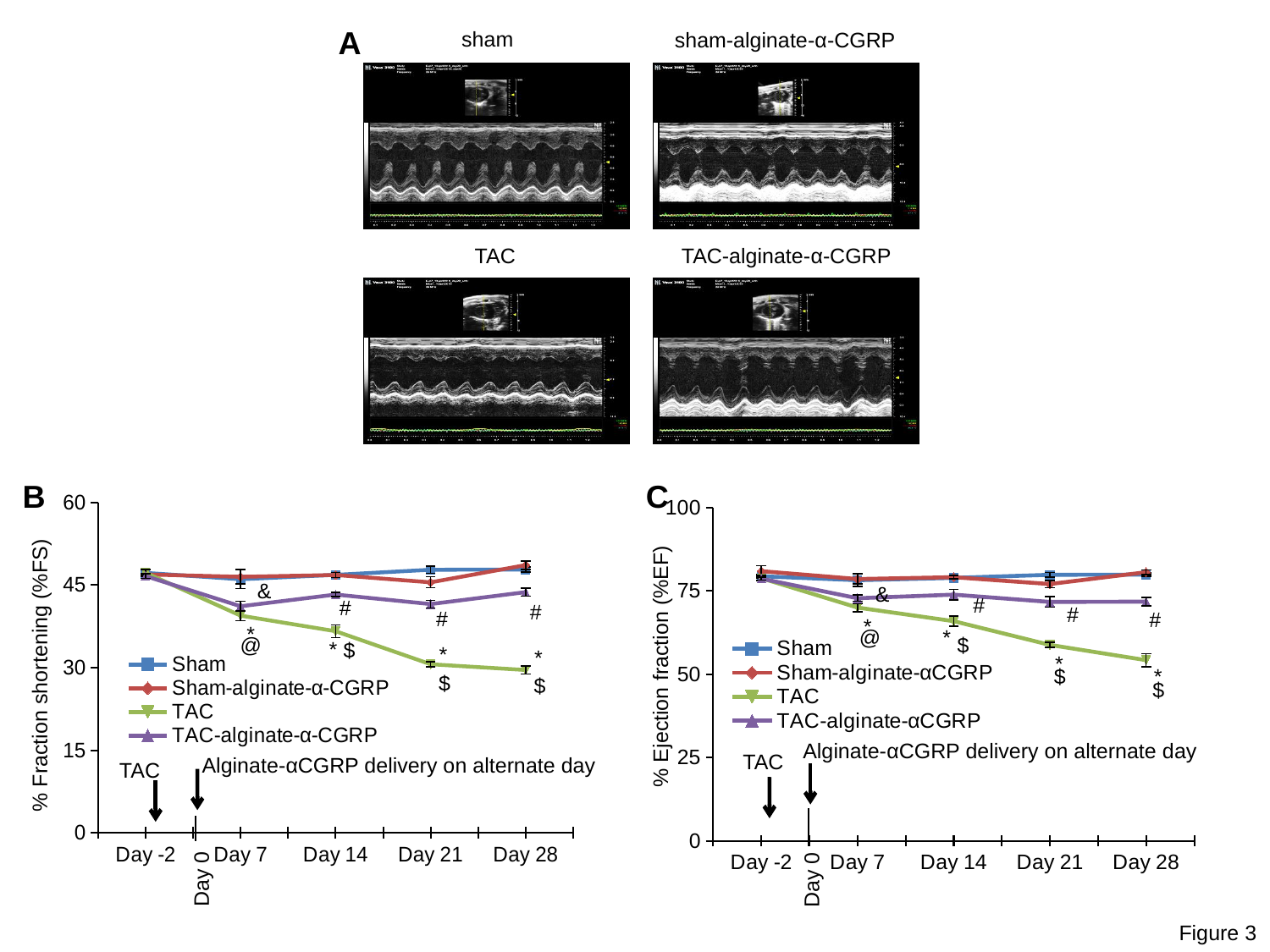

A
sham
sham-alginate-α-CGRP
TAC
TAC-alginate-α-CGRP
B
C
### Chart
| Category | Sham | Sham-alginate-αCGRP | TAC | TAC-alginate-αCGRP |
|---|---|---|---|---|
| Day -2 | 79.37 | 80.91 | 78.8 | 78.64 |
| Day 7 | 78.19 | 78.544 | 69.97 | 72.78 |
| Day 14 | 78.89 | 79.09 | 65.9 | 73.86 |
| Day 21 | 79.81 | 77.03 | 58.77 | 71.66 |
| Day 28 | 79.86 | 80.6 | 54.19 | 71.76 |
### Chart
| Category | Sham | Sham-alginate-α-CGRP | TAC | TAC-alginate-α-CGRP |
|---|---|---|---|---|
| Day -2 | 47.21910342857143 | 46.9389 | 47.23778571428572 | 46.636908 |
| Day 7 | 46.07193166666667 | 46.51643333333334 | 39.4612865 | 41.157847 |
| Day 14 | 46.86885766666666 | 46.841735 | 36.62413585714285 | 43.289635 |
| Day 21 | 47.79254 | 45.509162 | 30.625415285714286 | 41.55704871428571 |
| Day 28 | 47.817929750000005 | 48.636348 | 29.5833158333333 | 43.727358125 |&
&
#
#
#
#
#
#
*
*
@
*
$
@
*
$
*
*
% Ejection fraction (%EF)
*
% Fraction shortening (%FS)
*
$
$
$
$
Alginate-αCGRP delivery on alternate day
TAC
Alginate-αCGRP delivery on alternate day
TAC
Day 0
Day 0
Figure 3

## Slide 4
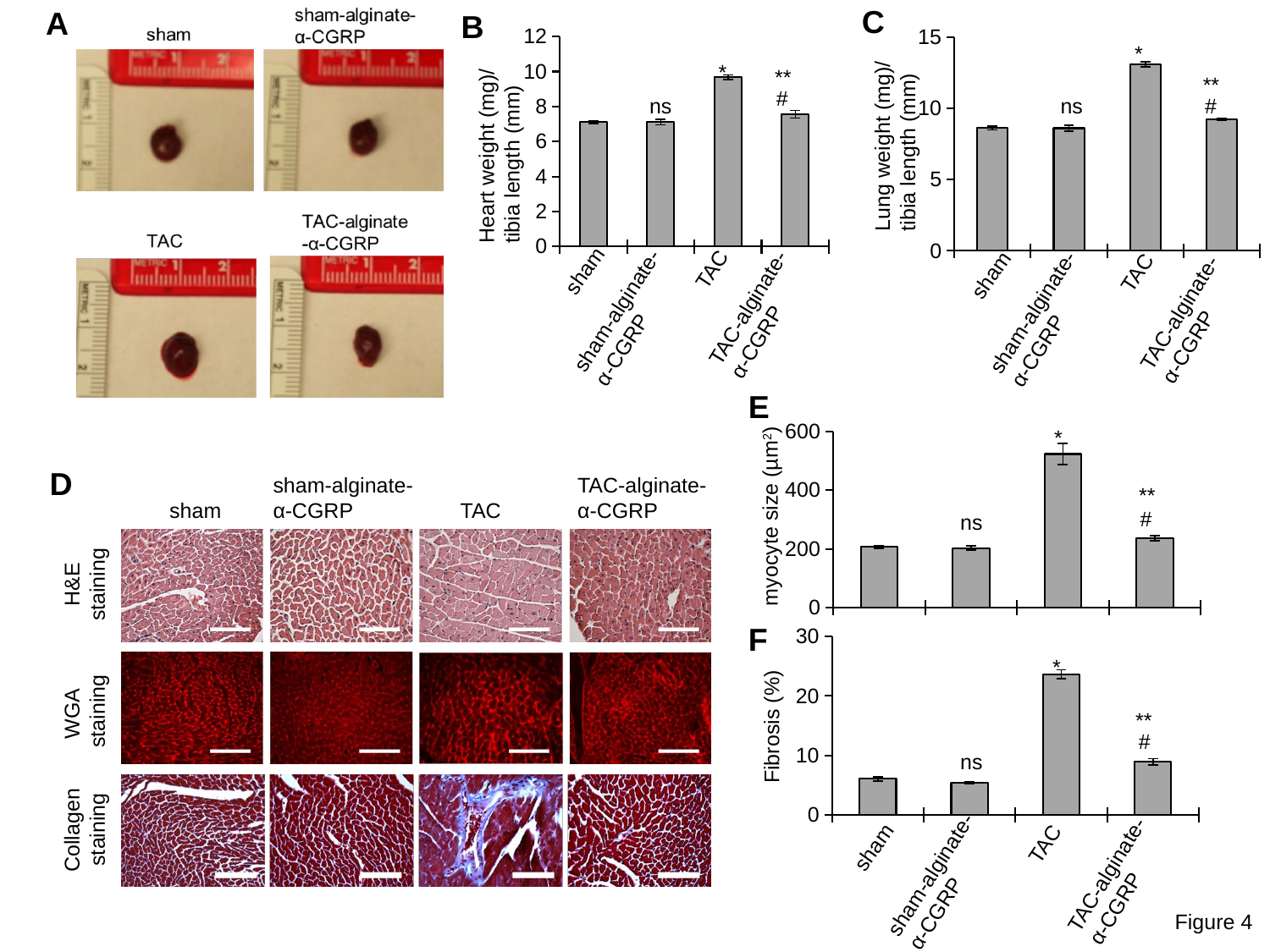

C
### Chart
| Category | |
|---|---|*
**
#
ns
Lung weight (mg)/
tibia length (mm)
TAC
sham
sham-alginate-
α-CGRP
TAC-alginate-
α-CGRP
A
B
### Chart
| Category | |
|---|---|*
**
#
ns
Heart weight (mg)/
tibia length (mm)
TAC
sham
TAC-alginate-
α-CGRP
sham-alginate-
α-CGRP
E
### Chart
| Category | |
|---|---|*
**
myocyte size (µm2)
#
ns
F
### Chart
| Category | |
|---|---|*
**
Fibrosis (%)
#
ns
TAC
sham
TAC-alginate-
α-CGRP
sham-alginate-
α-CGRP
D
sham-alginate-
α-CGRP
sham
TAC
H&E
staining
WGA
staining
Collagen
 staining
TAC-alginate-
α-CGRP
Figure 4

## Slide 5
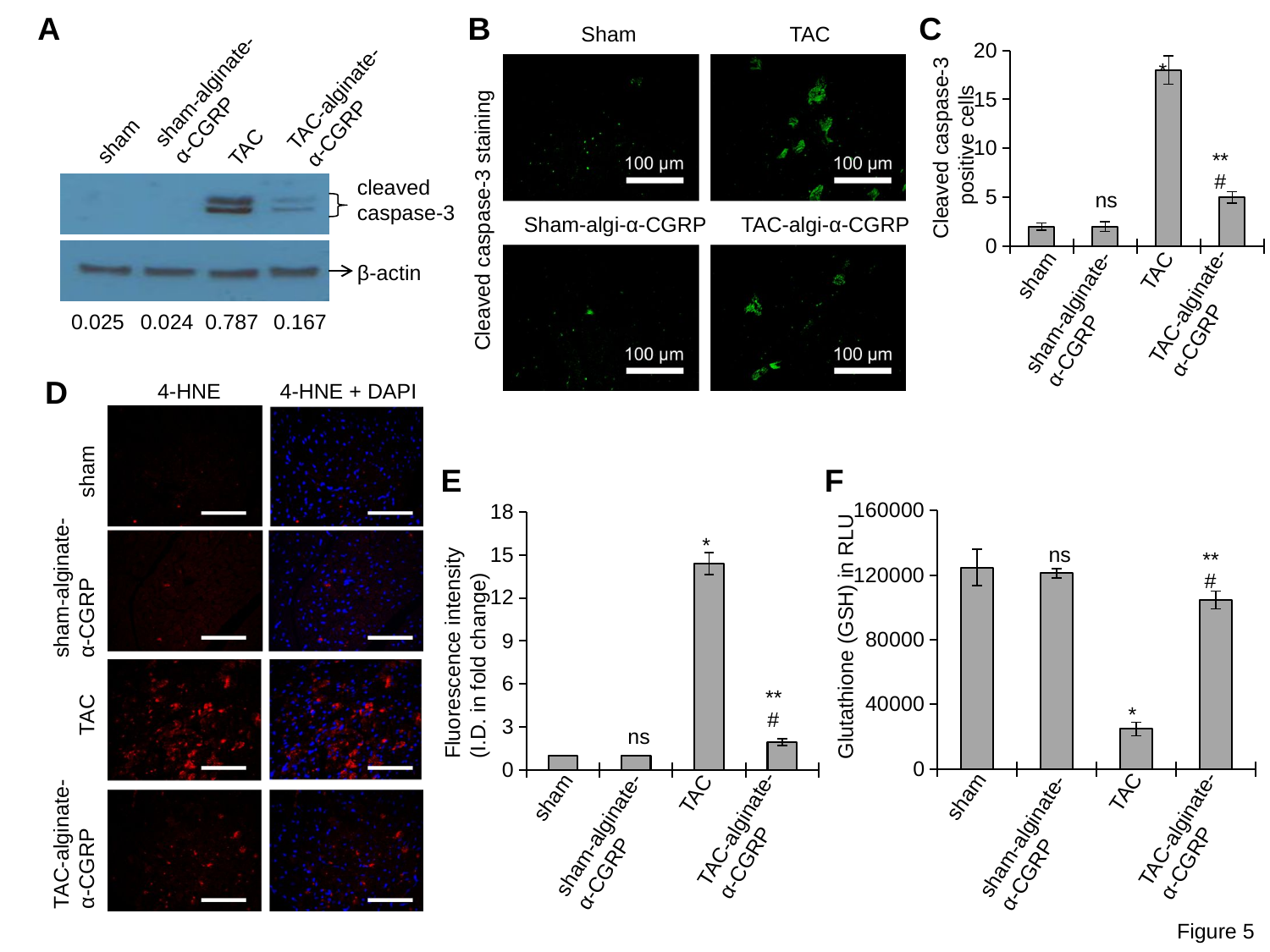

A
sham-alginate-
α-CGRP
TAC-alginate-
α-CGRP
sham
TAC
cleaved
caspase-3
β-actin
0.025
0.024
0.787
0.167
B
C
### Chart
| Category | |
|---|---|*
Cleaved caspase-3
positive cells
**
#
ns
TAC
sham
TAC-alginate-
α-CGRP
sham-alginate-
α-CGRP
Sham TAC
Cleaved caspase-3 staining
Sham-algi-α-CGRP TAC-algi-α-CGRP
D
4-HNE 4-HNE + DAPI
sham
sham-alginate-
α-CGRP
TAC
TAC-alginate-
α-CGRP
E
### Chart
| Category | |
|---|---|*
Fluorescence intensity
(I.D. in fold change)
**
#
ns
TAC
sham
TAC-alginate-
α-CGRP
sham-alginate-
α-CGRP
F
### Chart
| Category | |
|---|---|ns
**
#
Glutathione (GSH) in RLU
*
TAC
sham
TAC-alginate-
α-CGRP
sham-alginate-
α-CGRP
Figure 5

## Slide 6
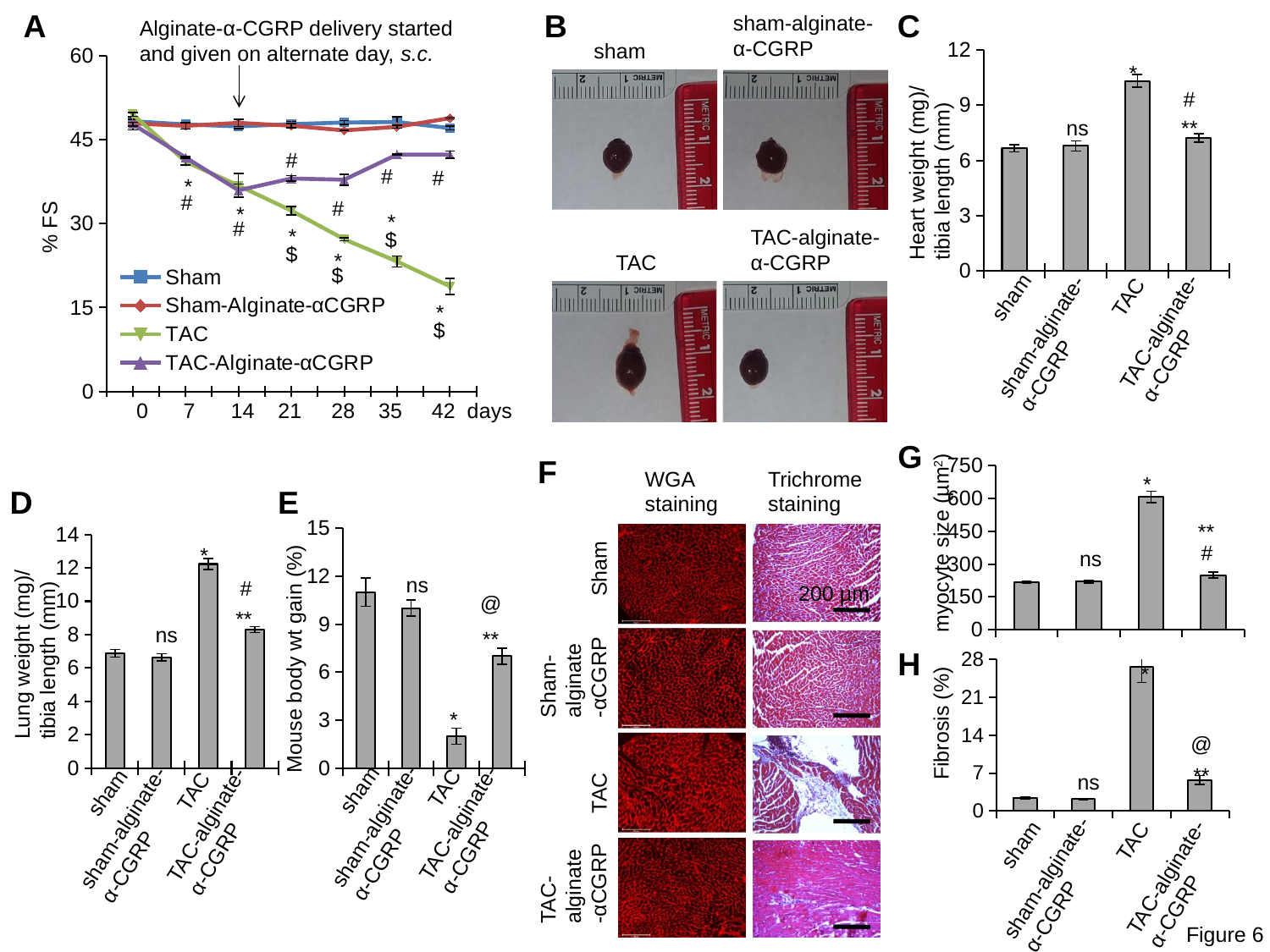

### Chart
| Category |
|---|C
### Chart
| Category | |
|---|---|*
#
ns
**
Heart weight (mg)/
tibia length (mm)
TAC
sham
TAC-alginate-
α-CGRP
sham-alginate-
α-CGRP
A
Alginate-α-CGRP delivery started
and given on alternate day, s.c.
### Chart
| Category | Sham | Sham-Alginate-αCGRP | TAC | TAC-Alginate-αCGRP |
|---|---|---|---|---|#
#
#
*
#
#
*
*
% FS
#
*
$
$
*
$
*
$
0 7 14 21 28 35 42 days
B
sham-alginate-
α-CGRP
sham
TAC-alginate-
α-CGRP
TAC
G
### Chart
| Category | |
|---|---|*
**
myocyte size (µm2)
#
ns
H
### Chart
| Category | |
|---|---|*
Fibrosis (%)
@
**
ns
TAC
sham
TAC-alginate-
α-CGRP
sham-alginate-
α-CGRP
F
WGA
staining
Trichrome
staining
Sham
200 µm
Sham-
alginate
-αCGRP
TAC
TAC-
alginate
-αCGRP
D
E
### Chart
| Category | |
|---|---|
### Chart
| Category | |
|---|---|*
ns
#
@
**
ns
**
Lung weight (mg)/
tibia length (mm)
Mouse body wt gain (%)
*
TAC
sham
TAC-alginate-
α-CGRP
sham-alginate-
α-CGRP
TAC
sham
TAC-alginate-
α-CGRP
sham-alginate-
α-CGRP
Figure 6
